# Disinfection By-Products and Congenital Anomalies: Evidence Still Inconclusive

**Published:** 2009-10

**Authors:** Angela Spivey

**Affiliations:** **Angela Spivey** writes from North Carolina about science, medicine, and higher education. She has written for *EHP* since 2001

Many observational studies have investigated a possible association between maternal exposure to mutagenic disinfection by‐products (DBPs) in the water supply and congenital anomalies in offspring, but literature reviews to date have shown the evidence to be inconclusive. Now researchers have reviewed newer epidemiologic studies that include more categories of anomalies, but again have found the evidence inconsistent **[*EHP*** **117:1486–1493; Nieuwenhuijsen et al.]**. They suggest several guidelines that would help future studies clarify this issue.

The authors reviewed all published epidemiologic studies that examined the association between congenital anomalies and exposure to DBPs, which form when organic matter in treated water reacts with chlorine disinfectant. The studies used a variety of indices of exposure, including the use of chlorination, DBP measurements in the public water supply, and information from participants about activities such as drinking, showering, and bathing.

When three or more studies evaluated the same exposure index and the same congenital anomalies, the authors performed a meta‐analysis to derive a summary risk estimate comparing high‐ and low‐exposure groups. When five or more studies investigated the relationship between total trihalomethane (TTHM) concentration and a specific anomaly, the authors conducted a meta‐analysis to arrive at exposure–response relative risk estimates per 10 μg/L TTHM. TTHM concentrations in local water sources have often been used in epidemiologic studies as a proxy for DBP exposure.

These meta‐analyses provide little evidence for an association. For all congenital anomalies combined, a statistically significant excess risk was found for high versus low exposure to chlorinated water or to TTHM, but the analysis was based on a small number of studies. The authors also found a statistically significant excess risk for ventricular septal defects, but that analysis included only three studies, and there was little evidence of an exposure–response relationship. In addition, the meta‐analyses were weakened by the fact that studies used very different exposure criteria. For instance, among studies that measured TTHM, one defined low exposure as lower than 60 μg per L water, whereas another defined high exposure as higher than 20 μg/L.

The authors point to the need for studies that take into account the complex mixtures of DBPs to which people are exposed, which can vary over time, by geographical area, and by route of exposure. This includes conducting studies in places with water supplies that have large amounts of specific DBPs, such as Barcelona, Spain, or Perth, Australia, which have high levels of brominated DBPs. Furthermore, showering, bathing, and swimming activities should be examined in more detail because these activities may yield different levels of exposure compared with drinking water exposure. The review also calls for studies that focus on specific congenital anomalies for which there is likely to be complete case diagnosis and reporting, in addition to case–control studies that follow up on possible associations with ventricular septal defects, which are particularly prone to under‐reporting in registry‐based studies.

